# Altered self-reported resting state mediates the effects of Mindfulness-Based Stress Reduction on mental health: a longitudinal path model analysis within a community-based randomized trial with 6-months follow-up

**DOI:** 10.3389/fpsyg.2023.1154277

**Published:** 2023-06-20

**Authors:** Lise Juul, Emilie Hasager Bonde, Lone Overby Fjorback

**Affiliations:** Department of Clinical Medicine, Danish Center for Mindfulness, Aarhus University, Aarhus, Denmark

**Keywords:** mental health (MeSH), mindfulness-based stress reduction (MBSR), mindfulness-based interventions (MBIs), mediation analysis (MeSH), default mode network (MeSH), resting state (MeSH)

## Abstract

**Background:**

A large body of randomized controlled trials (RCTs) has shown that mindfulness-based interventions are effective for improving mental health, but research is lacking in regards to the mechanisms of change. We aimed to investigate the mediating effects of self-reported altered resting state of Mindfulness-Based Stress Reduction (MBSR) on mental health, when provided as a universal intervention in a real-life context.

**Methods:**

Autoregressive path models with three time points of measurement, and contemporaneous and constant *b* paths were used in an RCT. The RCT took place in all five geographical regions of Denmark and included 110 schools and 191 schoolteachers. The schools were randomized 1:1 in each geographical region to intervention or a wait-list control group. The intervention was the standardized MBSR. Data were collected at baseline and after 3 and 6  months. The outcomes were perceived stress, measured by Cohen’s Perceived Stress Scale (PSS), symptoms of anxiety and depression, measured by Hopkins Symptom Check List-5 (SCL-5), and well-being measured by WHO-5 Well-being Index (WHO-5). The mediator was resting state measured by the Amsterdam Resting State Questionnaire (ARSQ).

**Results:**

Statistically significant mediated effects of altered ARSQ-subscales scores for Discontinuity of Mind, Planning, and Comfort were found for the MBSR effect on all outcomes; PSS, SCL-5 and WHO-5. Furthermore, statistically significant mediated effects of altered sleepiness subscale score of the effects on PSS and SCL-5 of MBSR were found. No statistically significant mediating effects of the subscales Theory of Mind, Self and Somatic Awareness for the MBSR intervention effect were found.

**Conclusion:**

The results support that the MBSR program can alter self-reported resting state, towards less mind wandering and more comfort, measured by the ARSQ, and that this may explain some of the mechanisms regarding the effectiveness of MBSR on mental health at 6 months, when provided as a universal intervention. The study provides insight into an active ingredient of how MBSR may improve mental health and well-being. It supports the suggestions that mindfulness meditation may be a sustainable way of training the mental health.

**Clinical trial registration:**ClinicalTrials.gov, identifier NCT03886363.

## Introduction

Mental health problems has been an increasing global public health concern during the past decades (Patel et al., 2018). The World Health Organization (WHO) describes mental health as “a state of mental well-being that enables people to cope with the stresses of life, realize their abilities, learn well and work well, and contribute to their community” (WHO, 2022a). Mental health is a complex continiuum and more than the absence of a mental disorder. Exposion to poverty, violence and inequality are known risk factors for poor mental health. Where as, social and emotional skills, positive social interactions, education, employment and safe neighbourhoods are well-known protective factors for good mental health (WHO, 2022a). Hence, mental health problems are complex public health issues that must be addressed both at system – and individual levels (Skivington et al., 2021; WHO, 2022a). There is a substantial need for knowledge on how to promote mental health and prevent mental illness in sustainable ways. Since mental health exists on a continuum, it is recommended not only to provide interventions to selected, high-risk groups, but also as universal interventions to the general population (Rose, 2008; Clifford Beers Foundation, M.H.W, 2012; Patel et al., 2018). It was recently again stated by WHO, that mental health promotion must be integrated in our everyday lives (WHO, 2022a).

Integrating of mindfulness in everyday life settings has been shown to have a positive effect on mental health; In a meta-analysis, van Agteren et al. (2021) found Mindfulness-based interventions (MBIs) to be among the most effective interventions in improving well-being when compared to other psychological interventions (Van Agteren et al., 2021). That was the case both when MBIs were provided to general populations as well as to selected groups with a mental illness (Van Agteren et al., 2021). Mindfulness has been defined as “…*the awareness that arises from paying attention on purpose in the present moment, non-judgementally, in the service of self-understanding, wisdom, and compassion*” (Kabat-Zinn, 2018). To support implementation of mindfulness in everyday life, the program Mindfulness-Based Stress Reduction (MBSR) was developed by Kabat-Zinn in 1979 (Kabat-Zinn, 1990). MBSR is distinct from conventional group therapy as it offers a practical educational firsthand approach on how to explore and understand the body, mind, and body–mind interactions. MBSR offers tools for training the mind and promoting mental health and resilience in everyday life (Kabat-Zinn, 1990). A large body of evidence from randomized controlled trials (RCTs) has documented positive effects of MBSR on well-being and symptoms of stress, anxiety and depression among various study populations with and without a clinical diagnosis, across the world (de Vibe et al., 2017). Hence, MBSR or adaptations of MBSR have shown effect in a range of diverse contexts. Even among asylum seekers with a severe trauma history and chronic postmigration stress, an adapted MBSR showed effects on symptom severity of posttraumatic stress disorder, depression, anxiety, and multimorbidity (Aizik-Reebs et al., 2021). The preventive effect of MBSR in non-clinical populations has also potential for public health (Khoury et al., 2015). Today, MBSR and other MBIs are widely used as mental health promotion intervention in, e.g., workplace contexts (Vonderlin et al., 2020), which is also recommended by the WHO (WHO, 2022b). However, knowledge on the mechanisms of change of MBSR is lacking (Dimidjian and Segal, 2015; Rosenkranz et al., 2019). To address public health problems, knowledge of mechanisms of change of complex interventions is highly important (Dimidjian and Segal, 2015; Rosenkranz et al., 2019; Skivington et al., 2021). This knowledge is especially beneficial for the design of future interventions and further for the transferability of the active intervention components across contexts (Skivington et al., 2021).

The neurobiological mechanisms of the effect of mindfulness on mental health are explained in the empirically based framework *“Self-awareness, -Regulation and – Transcendence (S-ART)”* (Vago and Silbersweig, 2012). The S-ART describes how mindfulness and compassion meditation practices have the potential to support neurocognitive processes both via top-down and bottom-up mechanishms and thereby improve mental health (Vago and Silbersweig, 2012). Top-down processing includes self-control and self-regulating (Wood and Smith, 2008) where impaired regulation is associated with addiction (Garland et al., 2013), distress, anxiety, rumination, and depression (Farb et al., 2007). Whereas bottom-up processing increases awareness of bodily and emotional signals, which are expected to reduce negative outcomes resulting from deficient self-control (Hölzel et al., 2011).

In line with the S-ART framework, Davidson (2021) have proposed that mental health is a trainable skill (Goleman, 2018; Davidson, 2021). By doing formal meditation practices, we can train our mental health condition like we can train our physical condition by doing physical exercises. Dahl et al. (2020) point to four core dimensions of well-being; awareness, connection, insight, and purpose, which can be promoted through mental health training (Dahl et al., 2020). The Awareness dimension enables one to direct and sustain attention, and awareness is crucial for mental health and the other dimensions. The Connection dimension refers to a subjective sense of giving and receiving care and reflects a willingness to engage in prosocial behaviours. The Insight dimension enables one to examine the implicit beliefs that inform self-related narratives. Self-inquiry is central to healthy psychological functioning, while automatic self-reflection that is overly negative, and self-critical is linked to mental illness (Philippi and Koenigs, 2014). The Purpose dimension fosters a self-perception that enables a person to embody their aspirations and values. Life aspirations enable individuals to organize and set goals, thus providing an overarching narrative that supports individuals in making sense of their lives and to stay motivated. A strong sense of purpose is associated with improved health outcomes and health behaviours (Alimujiang et al., 2019). The ability of people and societies to contribute to the world with a sense of meaning and purpose is associated with well-being (WHO, 2022c).

Distinct brain networks underlie our capacity to form and maintain well-being and healthy relationships (Dahl et al., 2020). The salience network is central in attention-regulation, emotion-regulation and in integrating interoceptive, automatic, and emotional information (Downar et al., 2016). The central executive network is associated with demanding cognitive task performance, and the default mode network is associated with reflection including values, purpose, and the longer term and ethical consequences of decisions and actions (Vago and Silbersweig, 2012; Immordino-Yang, 2016; Sezer et al., 2022). Unattended, these networks often operate unconsciously, allowing for the involvement of demanding cognitive tasks that are not aligned with one’s purpose. One may also be a prisoner of fear, anger, and delusions due to inappropriate or unskillsful thought patterns. The brain’s default network is spontaneously active, when we are not doing anything that requires focus and effort. Very often the default mode, also called resting state, is associated with mind wandering. Our automatic thoughts are often about what bothers, worries, and threatens us, largely because of our nervous system (our biological stress response) but also because of the culture, we are a part of (Goleman, 2018). Killingsworth and Gilbert (2010) showed that our human mind is occupied with mind wandering about half of our awake hours with consequences for our mental health (Killingsworth and Gilbert, 2010). When the brain engages in active tasks, the default mode activities calm down as those essential for these tasks gear up, but when the active tasks finish, the default mode activities ramp up again (Goleman, 2018). Therefore, many activities can quiet the default mode network and provide state effects. Mindfulness meditation may be understood as an activity that quiet the brain’s default mode network, although many novices find meditation challenging, e.g., due to mind wandering. However, sustained practice has the potential to cause a shift in the meditator’s relation to, e.g., thoughts and the self-narrative (Crane et al., 2017). The shift in perception may reduce the mind wandering and make more space for calmness and clarity about values and purpose (Goleman, 2018). Previous research has demonstrated that MBIs and meditation practices may alter brain function in neural regions and circuits that are essential for attention, interoception, emotion regulation, and self-relevant processing (Tang et al., 2015; Young et al., 2018). The MBSR program includes mindfulness meditation practices and other exercises that improve attention and emotion regulation and enhance sense of social connection and a less rigid sense of self by increasing insight (Rosenkranz et al., 2019). Hence, Rosenkranz et al. (2019) suggest altered resting state to be a plausible mechanism of change of the MBSR program on the effect on mental health.

**Figure 1:**
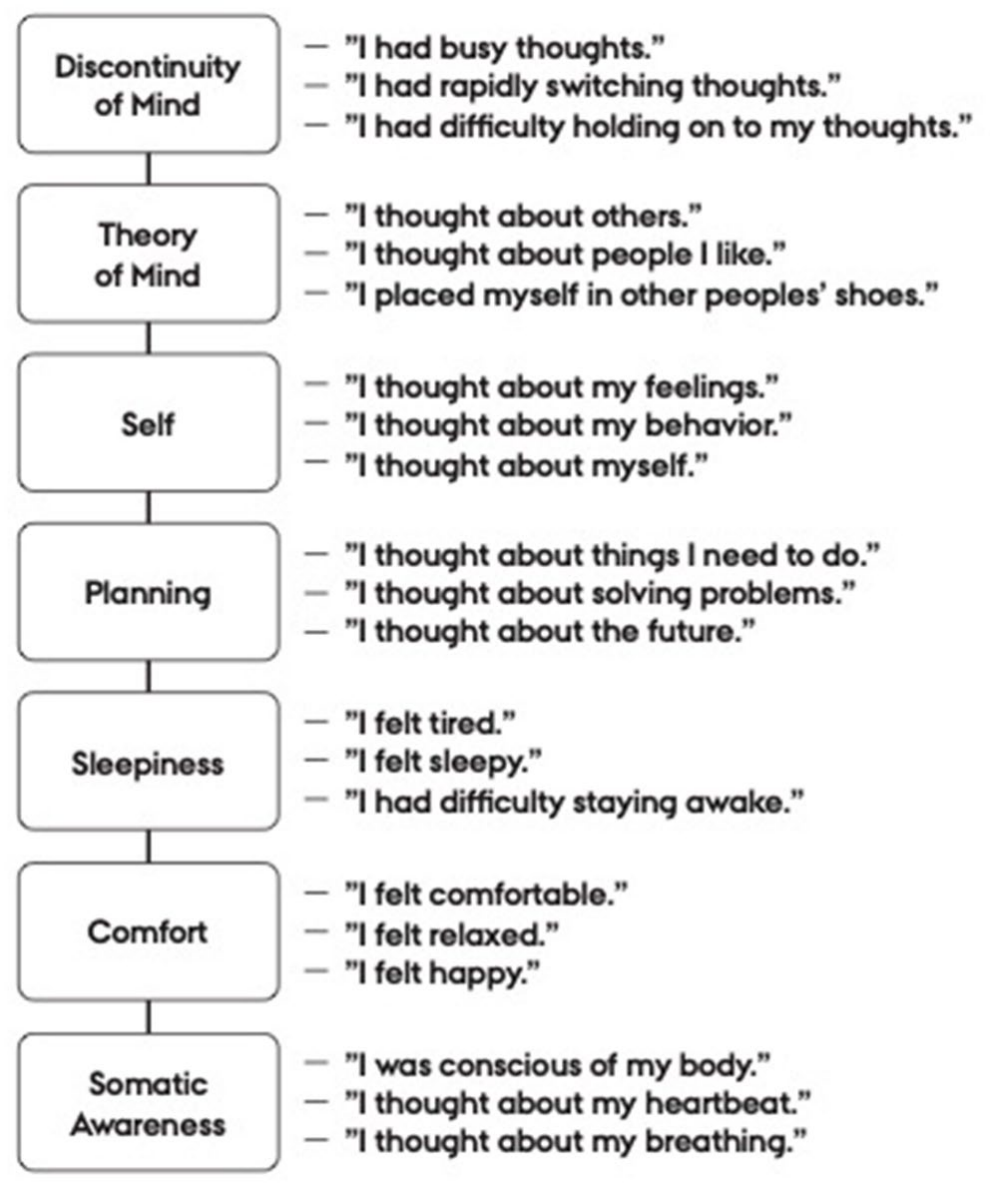
The Amsterdam Resting State Questionnaire subscales with respective items. From Diaz et al. (2013, 2014).

Resting state can be measured after a 5-min rest condition using the self-report Amsterdam Resting-State Questionnaire (ARSQ), which has shown to correlate with mental health and EEG markers (Diaz et al., 2013). The first ARSQ version included seven subscales as depicted in Figure 1 (Diaz et al., 2013, 2014). In a former study among young people, who had participated in MBSR, that was offered by their educational institution, we found that the large effect of MBSR on their mental health was associated with reduced mind wandering as measured by the subscale Discontinuity of Mind from ARSQ (Juul et al., 2021a). However, this study was limited by only having two measurement points and thereby did not comply with the requirement of temporal ordered measurements in mediation analyses (Goldsmith et al., 2017).

In the present study, the aim was to investigate the mediating effect of altered resting state measured by the ARSQ of the MBSR program on self-rated mental health in a community based RCT design with three repeated measurements. The RCT was conducted among Danish school teachers, who had participated in MBSR as part of a teacher-training program (Bonde et al., 2022). Our hypotheses were that the MBSR program would decrease mind wandering during rest and therefore decrease the Discontinuity of Mind and Planning, decrease Sleepiness, and increase Comfort and Somatic Awareness; and that these changes would improve mental health. We expected that MBSR could affect Self and Theory of Mind in both directions. They could decrease as a result of less mind wandering. MBSR could also increase the awareness of thoughts on self and others in the service of *self-understanding, wisdom, and compassion* as explained by the framework *Self-awareness, Regulation, −Tranformation (S-ART)*.

## Methods

### Design and participants

Longitudinal path models of RCT data were used. The study is a secondary mediation analysis of a published community-based RCT (Bonde et al., 2022). A two-arm parallel cluster-RCT was conducted using 110 schools as clusters in the research project *Stress-free Everyday LiFe for Children and Adolescents REsearch (SELFCARE)* (Juul et al., 2021b). Schools from all five geographical regions of Denmark were represented. A total of 68% of the included schools was municipal schools and 32% represented private schools. This distribution fairly represent the distribution of school types in Denmark (Juul et al., 2021b). A total of 191 schoolteachers were included. The characteristics of the participants are shown in Table 1 from Bonde et al. (2022).

**Table 1:** Characteristics of schoolteachers at baseline.

Characteristic	MBSR intervention (*n* = 97)		Wait-list control (*n* = 94)		Total (*n* = 191)	
	Included	Missing, *n* (%)	Included	Missing, *n* (%)	Included	Missing, *n* (%)
Sex, *n* (%)						
Men	10 (10.3)	0 (0)	6 (6.4)	0 (0)	16 (8.4)	0 (0)
Women	87 (89.7)	0 (0)	88 (93.6)	0 (0)	175 (91.6)	0 (0)
*Age, mean (SD), y*	46.2 (8.7)	0 (0)	44.2 (8.1)	0 (0)	45.2 (8.4)	0 (0)
*Self-reported mental health*						
*PSS, mean (SD)*	15.4 (5.4)	3 (3.1)	16.2 (6.0)	2 (2.1)	15.8 (5.7)	5 (2.6)
*SCL-5, mean (SD)*	1.9 (0.5)	1 (1.0)	1.9 (0.6)	1 (1.1)	1.9 (0.5)	2 (1.0)
*WHO-5, mean (SD)*	59.7 (16.9)	1 (1.0)	58.6 (17.1)	2 (2.1)	59.1 (17.0)	3 (1.6)
*BRS, mean (SD)*	4.3 (0.9)	2 (2.1)	4.3 (0.8)	2 (2.1)	4.3 (0.9)	4 (2.1)
*FFMQ-15, mean (SD)*	41.8 (5.5)	3 (3.1)	42.0 (5.7)	4 (4.3)	41.9 (5.6)	7 (3.7)
*ARSQ, mean (SD)*						
Discontinuity of Mind	9.0 (2.6)	1 (1.0)	9.0 (2.8)	4 (4.3)	9.0 (2.7)	5 (2.6)
Theory of Mind	8.6 (2.8)	3 (3.1)	9.2 (2.7)	3 (3.2)	8.9 (2.7)	6 (3.1)
Self	9.2 (2.3)	2 (2.1)	9.6 (2.0)	2 (2.1)	9.4 (2.2)	4 (2.1)
Planning	9.0 (2.9)	2 (2.1)	9.7 (2.9)	3 (3.2)	9.4 (2.9)	5 (2.6)
Sleepiness	6.6 (2.6)	4 (4.1)	6.4 (2.3)	3 (3.2)	6.5 (2.5)	7 (3.7)
Comfort	10.7 (1.9)	3 (3.1)	10.6 (2.0)	2 (2.1)	10.7 (2.0)	5 (2.6)
Somatic awareness	10.4 (2.2)	2 (2.1)	10.6 (2.3)	2 (2.1)	10.5 (2.2)	4 (2.1)

ARSQ, Amsterdam Resting-State Questionnaire; BRS, Brief Resilience Scale; FFMQ, Five Facet Mindfulness Questionnaire; MBSR, Mindfulness-based Stress Reduction; n, number; PSS, Cohen’s Perceived Stress Scale; SCL-5, The Hopkins Symptom Checklist 5; SD, standard deviation; WHO-5, WHO-5 Well-being Scale.

### Procedure and randomization

**Figure 2:**
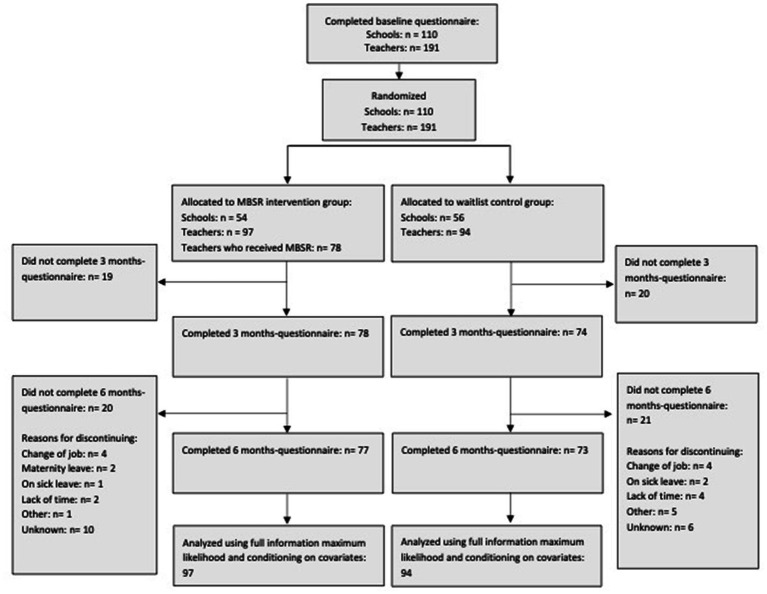
Flowchart for the trial.

For schools to enroll, the school principals had to provide consent for the teachers to participate in the trial and allow for trial participation during working hours. Recruitment was carried out between May 2018 and May 2019 through advertisements on Danish Center for Mindfulness (DCM)‘s webpage, social media posts, invitational letters to schools in selected regions, and local information meetings. Each school was allowed to enroll a maximum of three teachers. The individual schools chose how many and which teachers to include. Inclusion criterium for the teachers was an interest in becoming competent to teach mindfulness in schools. The teachers were informed that substance abuse problems and a schizophrenia diagnosis were not compatible with participation in the teacher training. All participants were informed about the trial and use of data. Teachers provided consent by completing the baseline questionnaire, in accordance with local legislation when conducting non-biological research (Datatilsynet, 2022). Data were collected and stored using Research Electronic Data Capture (REDCap) (Harris et al., 2009). Schools were randomized to begin teacher-training in 2019 or 2020. The randomization of schools was carried out as block-randomization and was performed in five blocks corresponding to geographical regions. For each region, a statistician received a list with anonymized school ids. The randomization was stratified by school size (more or less than 500 pupils), school type (private or municipal) and number of teachers included in the trial (1 or 2–3). The anonymous school ids were then linked to the schools’ identity. Randomization was conducted between February 2019 and September 2019 with a 1:1 allocation ratio. Figure 2 shows the flowchart for the trial (Bonde et al., 2022).

### Intervention group

Teachers representing schools randomized to the intervention group participated in the standardized MBSR program as part of teacher-training in 2019. MBSR was taught in-person in one group per region. The MBSR program consists of 8 weekly 2.5-h sessions and a 7-h silent retreat day. As an integral part of the MBSR program, participants were encouraged to actively practice mindfulness for 45–60 min a day 6 days a week. Two MBSR teachers delivered the MBSR courses in this trial. They were trained according to international standards (Kenny et al., 2020) and employed by The Danish Center for Mindfulness (DCM). However, these teachers were not part of the research group. The last author (LF) is trained and certified by University of Massachusetts and Brown University to train MBSR teachers. She supervised the MBSR teachers, which includes an inherent enhancement of intervention fidelity. The remaining parts of the teacher-training program were conducted after 6 months of follow-up. Thus, these the remaining parts of the teacher training program are outside the scope of the present study, and hence will not be elaborated on (Bonde et al., 2022). Regarding MBSR attendance, 78 (82%) of 97 eligible schoolteachers participated in an MBSR program (Figure 2). Of the 78 participants, all attended no less than five MBSR sessions with an average attendance of 7.6 sessions out of 9 (Bonde et al., 2022).

### Wait-list control group

The teachers representing schools randomized to the control group were put on a waiting list to receive the teacher-training program including an MBSR program, in 2020, i.e., after the 6-months follow-up (Bonde et al., 2022).

### Outcomes

All data for the present study were derived from questionnaire data that were measured at baseline, 3- and 6-months follow-up.

*The Perceived Stress Scale* (PSS) is a 10-item self-report measure of of perceived stress indicating how often respondents have experienced their life as unpredictable, uncontrollable, and overloaded in the past month (Cohen et al., 1983). The items are scored on a five-point Likert scale (total sum scores: 0–40), with higher scores indicating higher levels of perceived stress. Previous research has documented good validity and reliability of the scale (Cohen, 1988; Lee, 2012; Anita et al., 2015). Moreover, the scale has been translated to Danish and validated in Danish context (Eskildsen et al., 2015). Cronbach’s α was 0.87 in the present study sample.

*The Hopkins Symptom Check List-5* (SCL-5) is a 5-item measure of self-reported symptoms of anxiety and depression (Tambs and Moum, 1993). Scoring is performed on a four-point scale, ranging from 1 (not bothered at all) to 4 (extremely bothered). Avarage scores are calculated across the five items with higher scores indicating greater symptoms of anxiety and depression. Moreover, an SCL-5 score > 2 has been found predictive mental illness as assessed independently by psychiatrists (Strand et al., 2003). SCL-5 is a shortend version of the original 90-item SCL, which has been translated to Danish and validated in a Danish context (Olsen et al., 2004). The SCL-5 correlates with the 25-item SCL-anxiety and depression subscale at *r* = 0.92 (Strand et al., 2003). Cronbach’s α was 0.82 in the present study sample.

*The WHO-5 Well-being Index* (WHO-5) is a five-item self-report measure of subjective well-being. The items are designed to capture respondents well-being during the past 2 weeks. Each item is scored on a five-point scale. To calculate a total score, scores from each item are added and multiplied by four, resulting in a total score ranging from 0 to100 with higher scores indicating higher levels of well-being. A score below 50 indicates mental health problems (Bech et al., 2003). The WHO-5 has been translated into Danish (National Board of Health, 2021), and the scale is considered to be a valid measure of the overall well-being (Topp et al., 2015). Cronbach’s α was 0.83 in the present study sample.

Mediators were assessed using the *Amsterdam Resting State Questionnaire* (ARSQ). *ARSQ* is a self-report questionnaire, that samples thoughts and feelings during rest (i.e., an awake state characterized by the absence of goal-directed cognitive activity). The original ARSQ including 7 dimensions consists of 21 items scored on a Likert scale from 1 (completely disagree) to 5 (completely agree) after 5-min eyes-closed rest (Diaz et al., 2013, 2014). The items are depicted in Figure 1. The subscale scores are calculated as the mean score of the three items related to the subscale. Thus, subscales sum scores range from 3 to 15. Cronbach’s α were, in the present sample, for: Discontinuity of Mind: 0.78, Theory of Mind: 0.78, Self: 0.43, Planning: 0.74, Sleepiness: 0.77, Comfort: 0.69, and Somatic Awareness: 0.60. Researchers from DCM have translated ARSQ into Danish according to the WHO-guideline including forward- and expert panel back-translation (CourseHero, 2021). ARSQ has not been validated in a Danish context. However, previous Danish research studies have shown effect of MBSR on ARSQ dimensions (Juul et al., 2020, 2021a; Diachenko et al., 2021). In the present study, all subscales except from Self and Somatic Awareness had acceptable Cronbach’s *α*.

### Statistical methods

**Figure 3:**
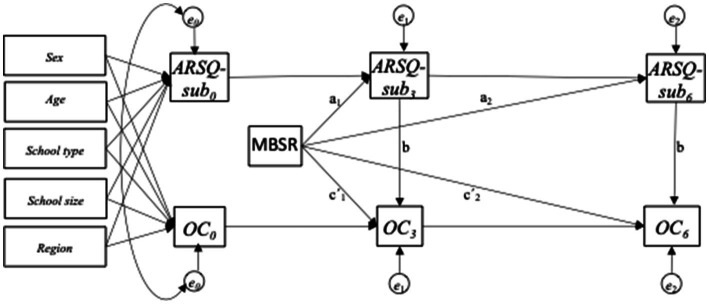
Mediation model.

Autoregressive models, with three time points of measurement, and contemporaneous and constant *b* paths were used (Mackinnon, 2008; Goldsmith et al., 2017) (see Figure 3). A total of 21 models each including a single ARSQ subscale, and an outcome were analyzed. The models were fitted in the structural equation model (SEM) framework, using full information maximum likelihood and conditioning on covariates to account for missing data under the missing at random assumption. Using SEM-models, the action theory, i.e., the relationship between MBSR and the ARSQ subscales, also termed the model’s *a* path, and the conceptual theory; the associations between the ARSQ subscales and the outcomes; the *b* paths, were analyzed simultaneously. The overall *a* path estimates were estimated by using all coefficients from paths from the MBSR program to the ARSQ subscale at 6 months. Baseline values of the ARSQ subscales and the outcomes were adjusted for the following covariates: sex, age, school type, school size and geographical region. We allowed for correlation between measurement errors for mediator and outcome at baseline.

To estimate the mediated effect of the MBSR program on outcomes at 6 months in the models, all paths that went from the MBSR program to outcome at 6 months through any measure of the mediator were identified first. Next, the coefficients within each of these paths were multiplied and added the path specific products to obtain the mediated effect. The unmediated effect was estimated based on paths from the MBSR program to outcome at 6 months not passing the mediator, i.e., all paths from the MBSR program going to outcomes at 6 months that started with a direct path from the MBSR program to outcome. The total effect equals the sum of all the mediated effects and the unmediated effect. The 95% Confidence Intervals (CI) of the estimates were estimated by use of 50 bootstrap replications. Goodness of fit of the models were tested by the widely used measures the comparative fit index (CFI) and the root mean squared error of approximation (RMSEA) (Acock, 2013). CFI compares the model with a baseline model that assumes no relationships among the variables. RMSEA considers the extent of error per degree of freedom and thereby take into account the complexity of models (Acock, 2013). The following criteria were used to evaluate model fit; A RMSEA below 0.08 indicates an acceptable model fit, and a RMSEA below 0.05 indicates good model fit. A CFI above 0.90 indicates a good model fit (Acock, 2013; Goldsmith et al., 2017; Loehlin, 2017). It indicates that a model does 90% better than a null model with the assumptions that the included variables are unrelated to each other (Acock, 2013).

The statistical package STATA 17 was used with all the analyses.

## Results

Results of the mediation models indicated that the MBSR program statistically significantly reduced discontinuity of mind, planning, and sleepiness during rest (Table 2). Furthermore, the MBSR program statistically significantly increased comfort and somatic awareness during rest. The MBSR program did not seem to have effect on thoughts about others and self during rest. The *b* path estimates indicated that all ARSQ subscales were statistically significant associated with PSS, e.g., that every 1-point increase in the Discontinuity of Mind scale was associated with an expected increase in PSS of 0.63 score point (95% CI 0.43 to 0.83) (Table 2). Another example: every 1-point increase in the Comfort subscale was associated with an expected decrease in PSS of 0.94 score point (95% CI 0.68 to 1.19). Except from Theory of Mind and Somatic awareness, the ARSQ subscales were also statistically significant associated with SCL-5 (Table 2). Expect from Theory of Mind, Self and Sleepiness, the ARSQ subscales were statistically significant associated with WHO-5 (Table 2). Accordingly, we found statistically significant mediated effects of altered Discontinuity of Mind, Planning and Comfort for the effect of the MBSR program on all the outcomes PSS, SCL-5 and WHO-5. We also found statistically significantly mediated effects of altered Sleepiness subscale score of the effects on PSS and SCL-5 of the MBSR program. In the models with statistically significantly mediated effects, the percentage of mediated effect of the total effect ranged from 14 to 79%. The overall mediated effect of Sleepiness on PSS was −0.40 (95% CI -0.73 to −0.06) out of the total effect of the MBSR program −2.80 (95%CI -4.46 to −1.14) score points (14%); And the overall mediated effect of the Planning subscale on WHO-5 was 3.62 (95% CI 1.92 to 5.31) of the total effect 4.61 (−0.53 to 9.74), (79%) (Table 2). No statistically significantly mediating effects of the subscales Theory of Mind, Self and Somatic Awareness for the MBSR program effect were found. Overall, goodness-of-fit tests showed acceptable model fits (Table 2).

**Table 2:** Mediated and unmediated effects of resting state measured by Amsterdam-Resting-State-Questionnaire (ARSQ) of the Mindfulness-Based Stress Reduction (MBSR) program on mental health at 6-months follow-up in secondary schoolteachers (*n* = 191)^a^ Outcome (OC).

Model Amsterdam Resting State Questionnaire subscale (ARSQ-sub) Outcome at 6 months (OC_6_)	*a*-path (overall) MBSR- > − > ARSQ-sub_6_ estimate (95% CI), *p*-value^b^	*b-*path (constrained) ARSQ-sub- > OC estimate (95% CI), *p*-value^b^	Mediated effect MBSR- > ARSQ-sub- > OC_6_ (all paths including ≥1 ARSQ_sub measurement) estimate (95% CI), *p*-value^b^	Total effect estimate (95% CI), *p*-value^b^	% mediated effect of total effect in single model	RMSEA CFI
Discontinuity of Mind						
PSS	−0.93 (−1.63 to −0.22), 0.010	0.63 (0.43 to 0.83), <0.000	−0.97 (−1.50 to −0.43), <0.000	−2.84 (−4.51 to −1.18), 0.001	34	0.07 0.91
SCL-5	−0.94 (−1.64 to −0.24), 0.008	0.05 (0.03 to 0.07), <0.000	−0.08 (−0.12 to −0.04), <0.000	−0.16 (−0.36 to 0.04), 0.116	50	0.08 0.87
WHO-5	−0.94 (−1.64 to −0.24), 0.009	−1.58 (−2.19 to −0.97), <0.000	2.57 (1.03 to 4.09), 0.001	4.91 (−0.34 to 10.16), 0.067	52	0.08 0.87
Theory of Mind						
PSS	−0.78 (−1.59 to 0.04), 0.064	0.25 (0.04 to 0.46), <0.000	−0.30 (−0.67 to 0.08), 0.126	−2.77(−4.40 to −1.15), 0.001	11	0.08 0.84
SCL-5	−0.79 (−1.60 to 0.03), 0.060	0.02 (−0.00 to 0.04), 0.087	−0.02 (−0.05 to 0.01), 0.171	−0.16 (−0.36 to 0.04), 0.111	13	0.08 0.82
WHO-5	−0.79 (−1.60 to 0.03), 0.059	−0.50 (−1.11 to 0.12), 0.113	0.62 (−0.29 to 1.52), 0.182	4.91 (−0.27 to 10.11), 0.063	13	0.08 0.83
Self						
PSS	0.21 (−0.47 to 0.88), 0.551	0.39 (0.16 to 0.62), 0.001	0.01 (−0.34 to 0.35), 0.956	−2.76 (−4.42 to −1.09), 0.001	0.3	0.06 0.88
SCL-5	0.20 (−0.48 to 0.88), 0.563	0.03 (0.00 to 0.05), 0.024	−0.00 (−0.03 to 0.03), 0.997	−0.16 (−0.37 to 0.04), 0.116	0	0.07 0.81
WHO-5	0.19 (−0.49 to 0.87), 0.580	−0.66 (−1.35 to 0.03), 0.062	0.00 (−0.65 to 0.66), 0.994	4.92 (−0.30 to 10.14), 0.065	0	0.06 0.85
Planning						
PSS	−1.15 (−1.80 to −0.50), 0.001	0.49 (0.29 to 0.69), <0.000	−0.99 (−1.52 to −0.46), <0.000	−2.77 (−4.41 to −1.14), 0.001	36	0.07 0.87
SCL-5	−1.12 (−1.77 to −0.48), 0.001	0.04 (0.02 to 0.06), <0.000	−0.09 (−0.15 to –0.04), 0.001	−0.15 (−0.35 to 0.04), 0.122	60	0.08 0.84
WHO-5	−1.14 (−1.79 to −0.50), 0.001	−1.71 (−2.28 to −1.14), <0.000	3.62 (1.92 to 5.31), <0.000	4.61 (−0.53 to 9.74), 0.079	79	0.07 0.88
Sleepiness						
PSS	−0.85 (−1.64 to −0.06), 0.035	0.31 (0.11 to 0.50), 0.003	−0.40 (−0.73 to −0.06), 0.020	−2.80 (−4.46 to −1.14), 0.001	14	0.06 0.88
SCL-5	−0.86 (−1.65 to −0.08), 0.032	0.02 (0.01 to 0.04), 0.013	−0.03 (−0.06 to −0.00), 0.025	−0.16 (−0.36 to 0.04), 0.111	19	0.07 0.78
WHO-5	−0.86 (−1.65 to −0.07), 0.032	−0.38 (−0.98 to 0.23), 0.223	0.51 (−0.30 to 1.31), 0.216	5.02 (−0.23 to 10.27), 0.061	10	0.06 0.86
Comfort						
PSS	0.92 (0.16 to 1.69), 0.017	−0.94 (−1.19 to −0.68), <0.000	−1.06 (−1.81 to −0.32), 0.005	−2.99 (−4.64 to −1.33), <0.000	35	0.07 0.91
SCL-5	0.94 (0.18 to 1.70), 0.015	−0.09 (−0.11 to −0.06), <0.000	−0.10 (−0.17 to −0.04), 0.003	−0.18 (−0.38 to 0.029), 0.071	56	0.08 0.86
WHO-5	0.94 (0.18 to 1.70), 0.016	2.89 (2.15 to 3.63), <0.000	3.38 (1.07 to 5.69), 0.004	5.33 (0.11 to 10.5), 0.045	63	0.08 0.88
Somatic awareness						
PSS	0.86 (0.07 to 1.66), 0.034	−0.26 (−0.49 to −0.03), 0.029	−0.33 (−0.74 to 0.09), 0.123	−2.87 (−4.52 to −1.21), 0.001	11	0.07 0.85
SCL-5	0.87 (0.07 to 1.67), 0.033	−0.02 (−0.04 to 0.01), 0.148	−0.02 (−0.06 to 0.02), 0.288	−0.17 (−0.36 to 0.03), 0.097	12	0.08 0.81
WHO-5	0.86 (0.07 to 1.66) 0.034	0.81 (0.14 to 1.49), 0.018	1.06 (−0.13 to 2.24), 0.080	5.19 (−0.059 to 10.44), 0.053	20	0.08 0.84

At baseline, residual covariance between all mediators and outcomes.

a According to Figure 3.

b Adjusted for sex, age, school type, school size and geographical region.

## Discussion

Mental health problems are complex public health issues that must be addressed both at system – and individual levels (Skivington et al., 2021; WHO, 2022a). There is no one single solution. However, evidence-based mindfulness training may be a part of the solution as it has shown to make a difference for mental health in diverse contexts (Khoury et al., 2015; de Vibe et al., 2017; Vonderlin et al., 2020; Aizik-Reebs et al., 2021). In our study context, MBSR was used as an universal mental health promoting intervention integrated in continuing education. The current study addressed the need of knowledge on the mechanisms of change of the MBSR program.

### Reductions in distracting thoughts and planning during rest, and an increase in comfort during rest were statistically significant mediators of the positive effects on mental health of the MBSR program at 6  months

Our results support the notion that the MBSR program can alter self-reported resting state measured by the ARSQ, and that this can explain important parts of the effect on mental health of the MBSR program at 6 months among Danish school teachers, who participated in the MBSR program as a part of a teacher-training (Table 2). More specifically, we found a reduction in distracting thoughts and planning during rest, and an increase in comfort during rest as statistically significant mediators of the positive effects on perceived stress, symptoms of anxiety and depression, and well-being of the MBSR program at 6 months. Less sleepiness also appeared to explain smaller parts of the effect of the MBSR program on reduced perceived stress, and symptoms of anxiety and depression. Less mind wandering, especially less planning during rest, seemed to explain large parts of the effects of the MBSR program on mental health, (e.g., 79% of the effect on well-being). Feeling comfort during rest also seemed to explain important parts of the effects of the MBSR program on improved mental health at 6 months. These results are in accordance with the results by Killingworth and Gilbert, who concluded that mind wandering was associated with decreased well-being (Killingsworth and Gilbert, 2010).

### No support for change in the amount of thoughts about others and self during rest as mediators of the effect of the MBSR program in this context

We found no support for the subscales Theory of Mind and Self as mediators of the effect of the MBSR program in this context. These subscales measure the “amount” of thoughts of others and self during rest and not the content and quality of the thoughts as such. Qualitatative studies are needed in order to achive more knowledge regarding this. The purpose of mindfulness meditation is not to have no thoughts and feelings. On the contrary, mindfulness meditation is an act of being present and becoming *aware* of one’s thoughts, feelings, behaviour, and self (Vago and Silbersweig, 2012). The key is to know when it is time to reflect, and when it is time to act. Research suggests that the brain either reflects (Default Mode Network) or acts (Executive Control Network) and the Salience Network is shifting between the Default Mode Network and the Executive Control Network (Sezer et al., 2022). It is for future studies to detect whether the practice of mindfulness may in fact enhance one’s ability to have some control over one’s inner life and ability to control each network. An fMRI-study compared a Mindfulness-Based Cognitive Therapy (MBCT) program with treatment as usual and found that the MBCT program led to decreased salience network connectivity to the lingual gyrus during a ruminative state (van der Velden et al., 2023). The study further showed that this change in salience network connectivity mediated improvements in the ability to sustain and control attention to body sensations (van der Velden et al., 2023). In the current study, we did find increased somatic awareness and positive associations of somatic awareness with perceived stress and well-being. However, it was not strong enough to serve as a statistically significant mediator in the present context.

### Mindfulness meditation may be a sustainable way of training the mental health

Our findings, that the MBSR program can change self-reported resting state and explain some of the effect on mental health after 6 months, when offered to a general population in a work-related context, support the assumption that mental health is a trainable skill (Dahl et al., 2020). The changes in resting state, including less mind wandering and more comfort, may make it easier to be at rest. Increased comfort may also be a result of less rumination and more healthy reflection during resting state. Being able to reflect, relax, be happy, and experience comfort are important health skills. The default mode network is associated with reflection including inner values, sense of purpose and long-term ethical consequences of decisions and actions (Immordino-Yang, 2016). Living in concordance with one’s values and life goals is associated with sustainable motivation and good mental health (Sheldon and Elliot, 1999; Ryan and Deci, 2000; Alimujiang et al., 2019; WHO, 2022c). When practicing mindfulness meditation, one practices being in a kind relationship with oneself during rest (Kabat-Zinn, 1990). An outcome of mindfulness meditation is meta-awareness, which is an important resource, as it may provide insights and the possibility for change (Goleman, 2018; Dahl et al., 2020). By observing ones thoughts with a non-judgmental attitude, a shift in ones relation to ones thoughts may occur, which can change ones perception and thereby also the default mode of the brain (Goleman, 2018; Dahl et al., 2020).

### Future research should focus on mechanisms of change of distinct types of mental health promoting interventions

In a former trial, the effects of the MBSR program measured on self-reported mental health did not differentiate when comparing the MBSR program to an active control intervention consisting of physical activity. However, effects on biological outcome measuring inflammatory response differentiated between the two groups, in favor of the MBSR program (Rosenkranz et al., 2013). These results indicate that different kinds of interventions can increase self-reported mental health, but the underlying mechanisms may be distinct (Rosenkranz et al., 2019). An overview of systematic reviews on the effectiveness of physical activity interventions on mental health recently concluded positive effects (Singh et al., 2023). Other interventions including, e.g., social network, spending time in nature, engaging in art or culture have also been suggested to improve mental health (Santini et al., 2017, 2023). This points to the importance of mechanism research as emphasized by the new MRC framework for developing and evaluating complex interventions (Skivington et al., 2021). The mechanisms of complex interventions improving mental health may differ, and some mechanisms may perhaps be more sustainable than others. The mechanisms of complex interventions may be the most important knowledge for improving health in sustaniable ways.

In their systematic review on mechanisms of change of the MBCT and MBSR programs from 2017, Alsubaie et al. concluded a lack of methodological rigor in the mediation studies. For example, none of the included studies had more than two measurement points and thereby failed to adhere to the criterion of temporal sequencing of change (Alsubaie et al., 2017). Mediation analysis of the MBSR program adhering to this criterion is still rare. However, Lengacher et al. (2021) showed, in a mediation model with three measurement points, that some of the effect of the MBSR program on anxiety and fatigue at 6 and 12 weeks among women, who had survived breast cancer, were mediated through reductions in fear of cancer recurrence, scored as the amount of thinking about breast cancer recurrence (Lengacher et al., 2021). Hence, the mechanisms in that study is comparable with the present results.

### Strengths and limitations

To our knowledge, this is the first study with temporal ordered data to investigate altered resting state measured by ARSQ as a mediator of the effect of the MBSR program. We used rigorous mediation models suggested by Goldsmith et al. (2017). We assumed 50 bootstrapped samples to be sufficient for the estimation of valid standard errors and thereby confidence intervals. It is a strength that the mediation analysis was conducted in a RCT design. We did not collect baseline data on all potential factors which might have an influence on mental health, such as physical activity, social network, trauma history, spending time in nature, engaging in art or culture etc. (Santini et al., 2017, 2023; Singh et al., 2023). We assume these factors to be equally distributed in the randomization and control group, but we cannot ensure this. We can neither rule out the risk of mass significance as we analyzed many models. However, we found it most correct not to select parts of the original ARSQ. It is a strength for the generalizability of the findings that the study was conducted using data from a real-life community randomized trial. However, the study population was to some extent homogenious as it included school teachers, mainly women with a moderate stress level at baseline. This fact should be taken into consideration when generalizing the findings.

In accordance with our previous results, the effects of the MBSR program on symptoms of anxiety and depression as measured by SCL-5, and on well-being as measured by WHO-5 were not statistically significant (Table 2) (Bonde et al., 2022). The 95% confidence intervals indicated lack of statistical power, e.g., for the WHO-5 effect estimate showing the uncertainty of MBSR effect from a decrease in WHO-5 score by 0.34 point or an increase in the score by 10.16 points (Table 2). However, Kraemer et al. (2002) suggest that it makes sense to investigate mediating effects even without statistically significant total effects (Kraemer et al., 2002).

Our trial lacked an active control group, which is a limitation of our study. We cannot rule out that our findings of potential mechanisms of MBSR also are present for other complex interventions addressing mental health. Future research should include an active control arm, e.g., including physical activity, or engagement with nature, or art and culture.

## Conclusion

The study results support that the MBSR program can alter self-reported resting state, towards less mind wandering and more comfort at rest, measured by the ARSQ. This can explain important parts of the effectiveness of the MBSR program on mental health at 6 months, when provided as a universal intervention. The study provides insight into an active ingredient of how the MBSR program can improve mental health and well-being. It supports the suggestions that mindfulness meditation may be a sustainable way of training the mental health.

## Data availability statement

The raw data supporting the conclusions of this article will be made available by the authors, without undue reservation.

## Ethics statement

The studies involving human participants were reviewed and approved by The Central Denmark Region Committee on Health Research Ethics. The project is registered at Aarhus University’s record of processing activities under journal no. (2016–051-000001/1145). Aarhus University hereby confirms that under these conditions the project is compliant with the EU and national legislation on data protection. Written informed consent for participation was not required for this study in accordance with the national legislation and the institutional requirements.

## Author contributions

LJ and LF designed the study. EB prepared the dataset. LJ analyzed, interpreted the data, and drafted the article. EB and LF critically revised the article. All authors contributed to the article and approved the submitted version.

## Funding

The Danish Parliament, Ministry of Health (Case number: 1800332) funded the training of the school teachers. The research was funded by TrygFonden (ID: 151692).

## Conflict of interest

The authors declare that the research was conducted in the absence of any commercial or financial relationships that could be construed as a potential conflict of interest.

## Publisher’s note

All claims expressed in this article are solely those of the authors and do not necessarily represent those of their affiliated organizations, or those of the publisher, the editors and the reviewers. Any product that may be evaluated in this article, or claim that may be made by its manufacturer, is not guaranteed or endorsed by the publisher.
